# Facile Synthesis of N-Doped Graphene Quantum Dots as Novel Transfection Agents for mRNA and pDNA

**DOI:** 10.3390/nano11112816

**Published:** 2021-10-23

**Authors:** Minchul Ahn, Jaekwang Song, Byung Hee Hong

**Affiliations:** 1 Department of Chemistry, College of Natural Sciences, Seoul National University, Seoul 08826, Korea; mincheol@snu.ac.kr (M.A.); saver04@snu.ac.kr (J.S.); 2 BioGraphene Inc., Advanced Institute of Convergence Technology, Suwon 16229, Korea; 3 Graphene Research Center, Advanced Institute of Convergence Technology, Suwon 16229, Korea

**Keywords:** gene delivery, graphene quantum dots, mRNA, pDNA, transfection

## Abstract

In the wake of the coronavirus disease 2019 (COVID-19) pandemic, global pharmaceutical companies have developed vaccines for the severe acute respiratory syndrome coronavirus-2 (SARS-CoV-2). Some have adopted lipid nanoparticles (LNPs) or viral vectors to deliver the genes associated with the spike protein of SARS-CoV-2 for vaccination. This strategy of vaccination by delivering genes to express viral proteins has been successfully applied to the mRNA vaccines for COVID-19, and is also applicable to gene therapy. However, conventional transfection agents such as LNPs and viral vectors are not yet sufficient to satisfy the levels of safety, stability, and efficiency required for the clinical applications of gene therapy. In this study, we synthesized N-doped graphene quantum dots (NGQDs) for the transfection of various genes, including messenger ribonucleic acids (mRNAs) and plasmid deoxyribonucleic acids (pDNAs). The positively charged NGQDs successfully formed electrostatic complexes with negatively charged mRNAs and pDNAs, and resulted in the efficient delivery and transfection of the genes into target cells. The transfection efficiency of NGQDs is found to be comparable to that of commercially available LNPs. Considering their outstanding stability even at room temperature as well as their low toxicity, NGQDs are expected to be novel universal gene delivery platforms that can outperform LNPs and viral vectors.

## 1. Introduction

COVID-19, coronavirus disease 2019, has threatened global society, and various types of vaccines have been developed to overcome the pandemic [1,2,3,4,5,6]. Gene-based vaccines such as messenger ribonucleic acid (mRNA) with lipid nanoparticles (LNPs) or deoxyribonucleic acid (DNA) with viral vectors have been approved by the United States Food and Drug Administration (FDA) for clinical uses [7,8,9,10,11]. Some pharmaceutic companies have developed the vaccine platform based on mRNAs employing LNPs as a delivery platform, which exhibits a high prevention rate [12,13]; others use viral vectors to transfer spike proteins genes into cells, which are similar to Ebola vaccines [14,15]. The therapeutic strategy that uses genes such as mRNA or plasmid DNA (pDNA) is called gene therapy, which has drawn much attention because it easily expresses desired proteins in the body through their corresponding sequence information to be transcribed and translated into therapeutic proteins to fight various diseases [16,17,18]. 

The viral vectors such as adeno-associated virus (AAV) are the main platforms that deliver genes into cells with high transfection efficiency [19,20]. However, they have some drawbacks such as limited size of cavities, high immunogenicity, mutagenesis, and undesired inflammatory responses [21,22,23]. LNPs, cationic polymers, and inorganic nanoparticles also have been actively recruited as non-viral vectors to overcome such limitations of viral vectors [24]. In particular, LNPs have already been approved by the FDA and have been used to deliver doxorubicin or mRNA [25,26]. However, LNPs also have drawbacks such as short half-life in the body, harsh storage conditions for stability, and low loading efficiency [27,28]. Therefore, it is of great importance to develop alternative gene delivery platforms to overcome the above-mentioned disadvantages.

Many researchers have tried to transfect genes using carbon-based materials such as graphene oxides (GOs) and graphene quantum dots (GQDs) [29,30,31,32]. GOs are the oxidized form of graphene sheets with a size ranging from several hundred nanometers to several micrometers [33]. As GOs are soluble in water and can interact with various drugs and genes, they have been utilized as drug delivery and biosensor platforms. It was previously reported that GOs can be modified with polyethylene glycol (PEG), polyethyleneimine (PEI), or/and targeting moieties to deliver genes into a cell [32,34,35]. Although the GO platform shows a good transfection efficiency, their high toxicity has limited their actual in vivo applications.

On the other hand, GQDs with a size of 1~10 nm that are functionalized with hydroxyl, carbonyl, alcohol, carboxylic groups, etc. exhibit relatively low toxicity [36]. This enabled the various applications of GOs in nanomedicine and bio-imaging fields, taking advantages of unique biological, chemical, and optical properties, amphiphilicity, and high biocompatibility [37]. In our previous reports, we found that GQDs with hydrophobic cores and hydrophilic edges interact with α-synuclein fibrils and dissociated them into monomers [38]. Additionally, we showed that GQDs exhibit low cytotoxicity and do not affect microbiome environment when fed to mice via oral administration [39]. GQDs also can be loaded with various drug molecules and genes through physicochemical conjugation between their functional groups [40,41,42,43,44]. Like GOs, GQDs need additional functionalization to interact electrostatically or covalently with therapeutic molecules or genes [45,46]. However, their relatively complicated synthetic processes has been a hurdle for practical applications [47,48], and the covalent modifications of genes possibly lower the gene activities [49]. In addition, most of previous studies have been focused on the delivery of pDNAs [50,51,52,53] as the intracellular distribution of GQDs near the nucleus is supposed to be advantageous for pDNA transfections [54,55]. Recently, Ya et al. reported the first study on the delivery of mRNAs using GQDs [56]. However, in this case, the GQDs required post-modifications with PEI via covalent bonding catalyzed by ethylenediamine (EDA) and dicyclohexycarbodiimide (DCC), which resulted in lower transfection efficiency than that of LNPs. In addition, the PEI-modified GQDs were incapable of delivering other types of genes such as pDNA.

GQD is a promising drug delivery platform that can deliver a wide range of small molecules, peptides, and genes into cells. It was found that GQDs localize in the late endosome or the lysosomes around the Golgi apparatus and nucleus after incubating cells with GQDs, which indicated that the cellular uptake of GQDs happens via endocytosis [55,57,58,59]. Furthermore, GQDs entered cells through diverse pathways related to endocytosis like caveolae-mediated endocytosis after drug loading. It is well known that GQDs interact with various types of drug molecules like doxorubicin and curcumin through electrostatic interaction or π—π interaction, where the loaded drugs don’t affect cellular uptake efficiency [42,60,61].

Herein, we synthesized positively charged NGQDs as gene transfection agents, utilizing the microwave-assisted hydrothermal reactions between polyethyleneimine (PEI) and citric acid precursors. It was expected that the negatively charged mRNA and pDNA interact electrostatically with the positively charged NGQDs. Indeed, NGQDs showed an excellent transfection efficiency even at ~200 times lower concentrations than their CC50 (50% of cytotoxic concentration ~125 μg/mL). To the best of our knowledge, these are the first applications of the as-synthesized NGQDs capable of transfecting both mRNA and pDNA without additional chemical modifications, which is expected to enable the cost-efficient large-scale synthesis that is essential for future clinical applications.

## 2. Materials and Methods

### 2.1. Synthesis of NGQDs

200 mg of citric acid (Sigma Aldrich, St. Louis, MO, USA) and 50 mg of PEI (branched, Mw 1800, Polyscience, Warrington, UK) were added to 15 mL of distilled water. After 30 min of sonication, a transparent solution was placed into the center of a microwave (MWO-2027, 800 W, Midea, Foshan, China). 30 s of a microwave-assisted hydrothermal reaction was iterated about 10 times until the reaction solution turned yellow. The product solution was filtered with 200 nm and 20 nm disc filters (Anodisc^TM^, GE Healthcare Life Sciences, Chicago, IL, USA) and dialyzed for 5 days with a 3.5 kDa dialysis tube (Snakeskin^TM^, Thermo Fisher Scientific, Waltham, MA, USA) for removal of the remaining reactants. The finished solution was lyophilized for 2 days.

### 2.2. Characterization of NGQDs

The morphology and size distributions of NGQDs were analyzed with a Cs-corrected transmission electron microscope (Cs-TEM; JEM-ARM200F, JEOL Ltd., Tokyo, Japan). The zeta potential was measured by a zeta potential analyzer (Zetasizer NanoS, Malvern Instruments, Malvern, UK). The functional groups of NGQDs were characterized by Fourier transform infrared (FT-IR; Vertex-80V, BRUKER, Billerica, MA, USA) and X-ray photoelectron spectroscopy (XPS; AXIS-His, Kratos Analytical Ltd., Manchester, UK). A Raman spectrometer (in Via Raman microscope, Renishaw, Wotton-under-Edge, UK) was used to identify the D and G bands of the graphene in the NGQDs. The absorbance of the NGQDs was analyzed with an ultraviolet-visible (UV-Vis) spectrophotometer (S-3100, Scinco, Seoul, Korea). The emission spectra at various excitation wavelengths were acquired with a spectrofluorometer (FP-8300, Jasco Inc., Tokyo, Japan).

### 2.3. Loading Capacity

To analyze the ratio of NGQDs to genes, 100 ng of mRNA (CleanCap^®^ EGFP mRNA, TriLink Biotechnologies, San Diego, CA, USA) and pDNA (pcDNA3-EGFP, Addgene, Watertown, MA, USA), encoding green fluorescent protein (GFP), were added to various amounts (0, 0.5, 1, 2, 4 μg) of NGQDs in 20 μL of 1× phosphate buffer saline (PBS) solution. The loading process was executed in a 1 mL tube. After 1 h of incubation, the series of complexes were mixed with 4 μL of LoadingSTAR^TM^ (Dyne Bio Inc., Seongnam, Korea) and the mixtures were loaded on a 1% agarose gel. The loading capacity of NGQDs was determined by measuring the intensity of the bands derived from the remaining genes after agarose-gel electrophoresis (Mupid-2plus, ADVANCE, Tokyo, Japan) at 100 V for 30 min.

### 2.4. Cell Viability Assay

HeLa cells were seeded in a 96-well plate at a density of 5 × 10^3^ cells for 24 h before transfection. HeLa cells were then treated with various concentrations of NGQDs in complete media for 24 h. After removal of the media, the cells were washed with 1× PBS solutions and incubated in 90 μL of serum-free media with 10 μL of cell counting kit-8 (CCK-8) (Dojindo Molecular Technologies Inc., Rockville, MD, USA) solution. To evaluate the cell viability of the treated cells, the optical density of formazan salt was measured at 450 nm using a microplate absorbance reader (Synergy Mx, BioTek, Winooski, VT, USA), and the background absorbance of the media was subtracted. Experiments were carried out in triplicate.

### 2.5. Gene Transfections Efficiency

HeLa cells were seeded in a 24-well plate at a density of 3 × 10^4^ cells. After incubation for 24 h, the cells were treated with 1× PBS (Ctrl), mRNA, Lipofectamine^®^ 2000 (Thermo Fisher Scientific), NGQDs, mRNA with Lipofectamine^®^ 2000, and mRNA with NGQDs in 0.5 mL of serum-free media. For preparation of the complex-containing genes and the NGQDs, 3 μL of mRNA solution (10 μg/mL) and 6 μL of NGQDs solution (0.1 mg/mL) were mixed and 2 μL of 10× PBS solution was added with 9 μL of deionized water. After incubation with the complexes for 24 h, media were eliminated, and the cells were washed with 1 mL of 1× PBS solution. Bright-field and fluorescence images (λ_Ex_/λ_Em_ = 488 nm/507 nm) were acquired by fluorescence microscopy (Nikon Co., Tokyo, Japan) with Metamorph image analysis software (Molecular Devices, San Jose, CA, USA). For quantitative analysis of the transfection, cells were trypsinized and harvested, followed by suspension of the cells in 0.5 mL of 1× PBS solution. The fluorescence intensity of the samples was analyzed by flow cytometry (BD FACSLyric, BD Biosciences, New York, NY, USA).

## 3. Results and Discussion

**Figure 1:**
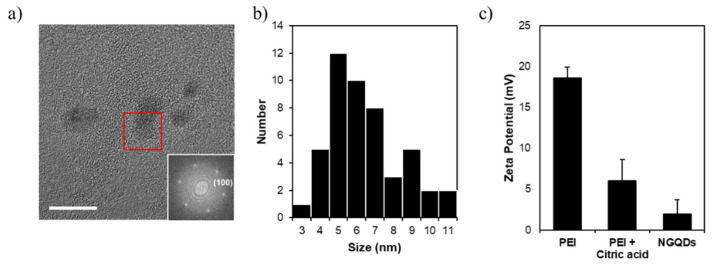
(**a**) TEM images of NGQDs (scale bar = 20 nm). (**b**) Size distribution of NGQDs. (**c**) zeta potentials of PEI, PEI + citric acid and NGQDs.

PEI and citric acid were recruited as precursors and GQDs were synthesized by the microwave-assisted hydrothermal reaction. The synthesized GQDs were named N-doped GQDs (NGQDs). TEM observation of prepared NGQDs was performed to analyze their morphology and size. The NGQDs were identified as nanoparticles with an overall diameter distribution of 3–11 nm and average of 7.03 nm ± 0.27 nm (Figure 1a,b). The zeta potential is measured to be 1.91 ± 1.77 mV, indicating that the surface charge of NGQDs in deionized water is considerably positive (Figure 1c), which allows NGQDs to interact electrostatically with genes that are negatively charged due to phosphate backbones.

**Figure 2:**
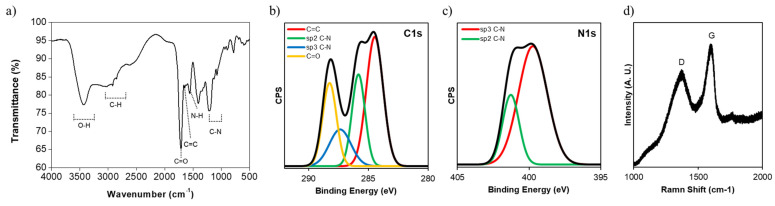
(**a**) FT-IR spectrum, (**b**,**c**) XPS spectra (C1s, N1s), and (**d**) Raman spectrum of NGQDs.

We performed FT-IR and XPS for the analysis of functional groups. We observed the representative peaks of the NGQDs at 1720 and 1650 cm^−1^, which were interpreted as the C=O stretching and C=C stretching vibrations in carboxylic acid and aromatic groups, respectively (Figure 2a). In addition, we could clearly observe the peaks at 1550 and 1000–1250 cm^−1^, which correlated with N-H bending in primary and secondary amine groups as well as the C-N bond of amine groups, and appeared in the FT-IR spectra of the precursors (Figure S1). In XPS C1s and N1s spectra, we found that NGQDs contain aromatic sp2 C=C, sp2 C-N, sp3 C-N, and carboxyl groups, which correspond to the peaks at 284.5, 285.7, 287.5, and 288.6 eV (Figure 2b,c). We also observed the D band and the G band at 1350 cm^−1^ and 1580 cm^−1^, respectively, as the characteristic peaks of graphene from the Raman spectrum (Figure 2d). These results indicate that the NGQDs are composed of hydrophobic aromatic sp^2^ domains and hydrophilic functional groups such as carboxylic acid, amine groups, etc.

**Figure 3:**
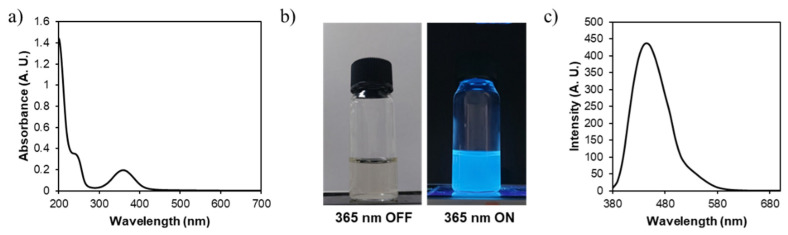
(**a**) UV-visible absorption spectrum, (**b**) optical and fluorescence (λ_Ex_ = 365 nm) images, and (**c**) emission spectrum of NGQDs (λ_Ex_ = 360 nm).

NGQDs show UV-Vis absorption peaks at 250 and 350 nm, which corresponded to the π–π* transition of aromatic sp2 C=C and the n–π* transition of carbonyl C=O [62], respectively, which means that NGQDs are composed of aromatic sp2 C=C domains and carbonyl groups (Figure 3a). In addition, the NGQDs emitted blue fluorescence (E_m, max_ = 432 nm) at 365 nm excitation (Figure 3b,c). From the overall characterization data, we demonstrated that NGQDs had the characteristic properties of GQDs: the graphitic core with the diverse functional groups and the positive charge required to interact with genes.

**Figure 4:**
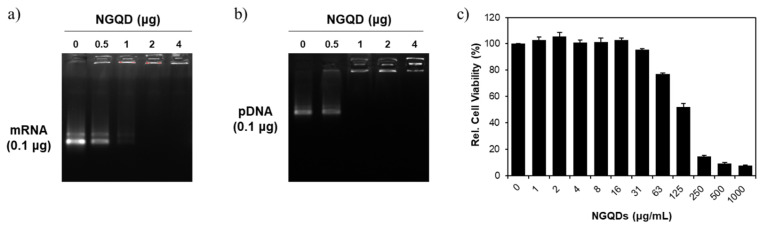
Loading capacity of NGQDs to (**a**) mRNA and (**b**) pDNA. (**c**) Relative cell viability of NGQDs.

Next, we performed loading tests to evaluate the loading capacity of NGQDs to genes. We mixed the NGQDs with two types of genes in 1× PBS solutions and incubated them at room temperature. According to the results from the agarose-gel electrophoresis, the columns for 1 μg and 0.5 μg NGQD, with respect to 0.1 μg mRNA and 0.1 μg pDNA in agarose gel, show an incomplete band shift. Thus, we supposed that the equivalent amount of NGQDs for perfect loading would be somewhere between 1 and 2 μg for 0.1 μg mRNA, and 0.5 and 1 μg for 0.1 μg pDNA, respectively (Figure 4a,b). The positively charged NGQDs could interact with genes via electrostatic force and formed complexes with the genes by the simple mixing at room temperature.

Prior to in vitro gene transfection with NGQDs, we verified the cytotoxicity of the NGQDs via a CCK-8 assay kit. For the CCK-8 assay, various concentrations of NGQDs from 1 μg/mL to 1000 μg/mL were treated to HeLa cells in complete media for 1 day. NGQDs exhibited a dose-dependent toxicity, and reduced cell viability was observed at a concentration of 63 μg/mL (Figure 4c).

**Figure 5:**
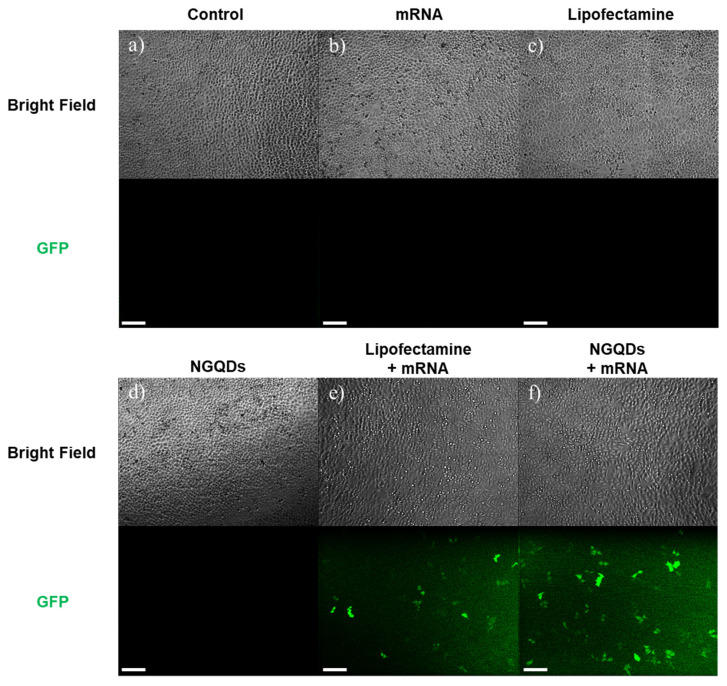
Fluorescence microscopy images of HeLa cells after 24 h transfection with (**a**) control (1 × PBS), (**b**) mRNA only, (**c**) Lipofectamine only, (**d**) NGQDs only, (**e**) Lipofectamine + mRNA complex, and (**f**) NGQDs + mRNA complex. All scale bars are 200 μm.

To evaluate the transfection efficiency of NGQDs, we delivered mRNA into HeLa cells with NGQDs. As shown in Figure 4a, the NGQDs formed complexes with mRNA at a 20:1 ratio (wt/wt), and the results show that 30 ng of mRNA, encoding a GFP, formed complexes with 600 ng of NGQDs before transfection. As a positive control, Lipofectamine 2000 was used, a representative transfection agent that forms liposomes with various genes. After incubation for 1 h at room temperature, the NGQDs + mRNA complexes, as well as other groups—1 × PBS (control), mRNA only, Lipofectamine only, NGQDs only, Lipofectamine + mRNA—were treated to HeLa cells for 24 h. To visually confirm the transfection by each group, we observed green fluorescence through fluorescence microscopy. The fluorescence images from Figure 5 show that the number of cells expressing GFP in the NGQDs + mRNA complex group was comparable to groups using Lipofectamine as a transfection agent (Figure 5e,f). These results indicate that the NGQDs with gene complexes had entered the cell successfully. This result shows that the NGQDs formed complexes with mRNA and pDNA at 10–20 times their amounts, which implies that several NGQDs wrap an mRNA or DNA molecule and deliver it to nearby cellular membranes, followed by cellular uptake. We supposed that the neutralized charge complex between NGQDs and genes enables cellular uptake, although some have previously reported the preferred interaction of the positively charged NGQDs with the cellular membrane [63,64,65]. Although the NGQDs had fluorescence emission at around 500 nm under excitation at 360 nm, a similar wavelength to GFP emission, the fluorescence in Figure 5f did not result from NGQD fluorescence. The fluorescence of NGQDs was not visualized in fluorescence microscope images because the excitation range of NGQDs is from 280 to 430 nm, and not the GFP excitation wavelength (~488 nm) (Figure 5d and Figure S2). From these results, we can conclude that NGQDs have been successfully delivered mRNA into cells, followed by translation to generate therapeutic proteins.

**Figure 6:**
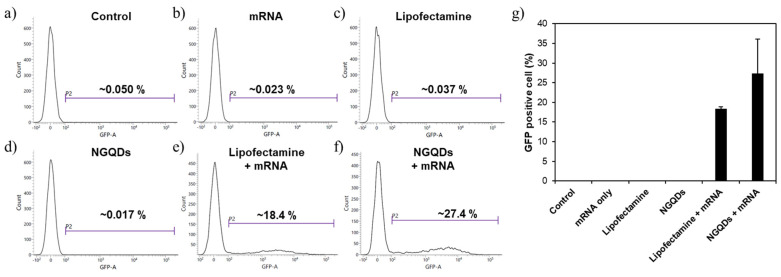
Flow cytometry analysis of HeLa cells after 24 h transfection with each group; (**a**) control, (**b**) mRNA only, (**c**) Lipofectamine only, (**d**) NGQDs only, (**e**) Lipofectamine + mRNA complex, (**f**) NGQDs + mRNA complex groups. (**g**) mRNA transfection efficiency of each group.

We performed a flow cytometry analysis to quantify the transfection efficiencies of each group. Similar to the results of fluorescence microscopy, GFP-expressing cells were not detected in control, mRNA only, Lipofectamine only, and NGQDs only groups. In particular, the fluorescence of NGQDs at around 500 nm did not affect the flow cytometry analysis. Although both the NGQDs + mRNA complex and the Lipofectamine + mRNA complex had similar fluorescence images, the NGQDs + mRNA complex showed enhanced transfection of up to 50% when compared to the Lipofectamine + mRNA complex, the positive control in the quantitative analysis (Figure 6 and Figure S3). Through these experiments, NGQDs have great potential as mRNA delivery platforms for vaccinations or gene therapy even though there are a few more factors to be validated, such as in vivo safety and efficiency.

**Figure 7:**
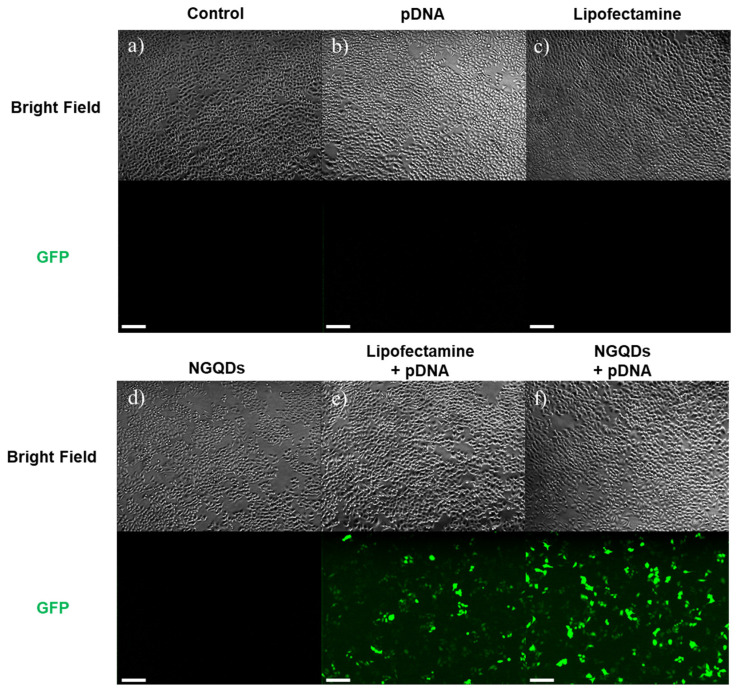
Fluorescence microscopy images of HeLa cells after 24 h transfection with (**a**) control, (**b**) pDNA only, (**c**) Lipofectamine only, (**d**) NGQDs only, (**e**) Lipofectamine + pDNA complex, and (**f**) NGQDs + pDNA complex. All scale bars are 200 μm.

In addition to mRNA, we performed a pDNA transfection test using NGQDs. similarly, the GFP-encoding pDNA complexed with NGQDs were prepared by simply mixing them at room temperature. After incubation for 1 h, we treated each group to HeLa cells for 24 h. Fluorescence microscopy images after treatments show that NGQDs had the best performance as pDNA delivery platforms. In the fluorescence microscope image, the NGQDs + pDNA group showed the strong green fluorescence comparable to the Lipofectamine + pDNA group (Figure 7).

**Figure 8:**
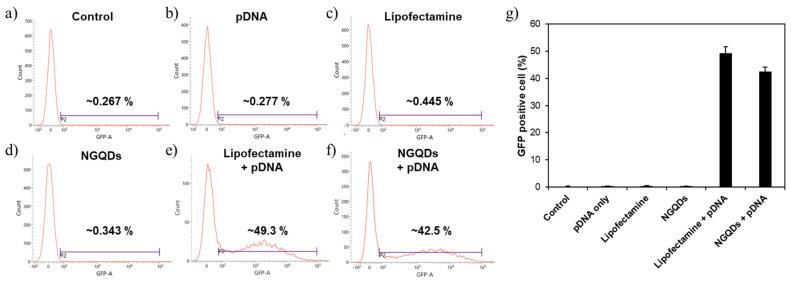
Flow cytometry analysis of HeLa cells after 24 h transfection with each group; (**a**) control, (**b**) pDNA only, (**c**) Lipofectamine only, (**d**) NGQDs only, (**e**) Lipofectamine + pDNA complex, (**f**) NGQDs + pDNA complex groups. (**g**) pDNA transfection efficiency of each group.

A flow cytometry analysis was performed to quantify the transfection efficiencies. As a result of the flow cytometry analysis, the transfection efficiency of NGQDs (~42.5%) was slightly lower than that of the Lipofectamine + pDNA group (~49.3%) (Figure 8 and Figure S4). The disagreement between the flow cytometry results in Figure 8g and the fluorescence microscopy image in Figure 7e,f is likely due to the difference in the fluorescence intensities of the transfected cells. The cells treated with pDNA + NGQDs tend to express strong fluorescence in flow cytometry analysis, which made the fluorescence image brighter as shown in Figure 7e,f. Despite that mRNA and pDNA work at the different intracellular locations, we confirmed that NGQDs are capable of delivering both genes into the cells, and the transfected genes are functioning, successfully [54]. 

## 4. Conclusions

In conclusion, we synthesized positively charged NGQDs to deliver genes such as mRNA and pDNA. NGQDs were synthesized using PEI and citric acid as precursors to give positive charges. The NGQDs synthesized via microwave-assisted hydrothermal reactions were characterized by TEM, DLS, FT-IR, XPS, and Raman spectroscopy. Overall characterization data exhibit that NGQDs consist of a hydrophobic graphene domain and hydrophilic functional groups such as carboxylic acid and amine. It is confirmed that the positively charged NGQDs interact with the model mRNA and pDNA, the representative types of components for gene therapy, and transfect the cells, successfully. Thegene transfection efficiency of NGQDs was measured to be comparable to Lipofectamine that is recognized as the “gold-standard” for in vitro gene transfection agents. Even in the case of mRNA transfection, the NGQDs exhibited a better performance than Lipofectamine. We expect that NGQDs can be utilized in the clinical field after further studies on their toxicity and metabolism in consideration of the previous studies on the intracellular distribution of NGQDs [55,66].

## Data Availability

The data presented in this study are available on request from the corresponding author.

## Supplementary Materials

The following are available online at https://www.mdpi.com/article/10.3390/nano11112816/s1, Figure S1: FT-IR spectra for NGQDs, PEI + citric acid, and PEI. Figure S2: Emission spectra of NGQDs at excitation wavelength from 280 nm to 580 nm. Figure S3: Flow cytometry analysis for mRNA transfection efficiency. Figure S4: Flow cytometry analysis for pDNA transfection efficiency.
