# Supplementary material for: Facile Synthesis of N-Doped Graphene Quantum Dots as Novel Transfection Agents for mRNA and pDNA
Source: Nanomaterials (Basel). 2021 Oct 23;11(11):2816. doi: 10.3390/nano11112816 (PMC8620666; doi:10.3390/nano11112816)
Supplement: Supplementary file 1 [file nanomaterials-11-02816-s001.zip › nanomaterials-1370787-supplementary.pdf]

nanomaterials

Supplementary Materials

Facile Synthesis of N-Doped Graphene Quantum Dots as Novel Transfection Agents for mRNA and pDNA

Minchul Ahn [1,3], Jaekwang Song [1] and Byung Hee Hong [1,2,*]

[1] Department of Chemistry, College of Natural Sciences, Seoul National University, Seoul 08826, Korea; mincheol@snu.ac.kr (M.A.); saver04@snu.ac.kr (J.S.)
[2] Graphene Research Center, Advanced Institute of Convergence Technology, Suwon 16229, Korea
[3] BioGraphene Inc., Advanced Institute of Convergence Technology, Suwon 16229, Korea
* Correspondence: byunghee@snu.ac.kr

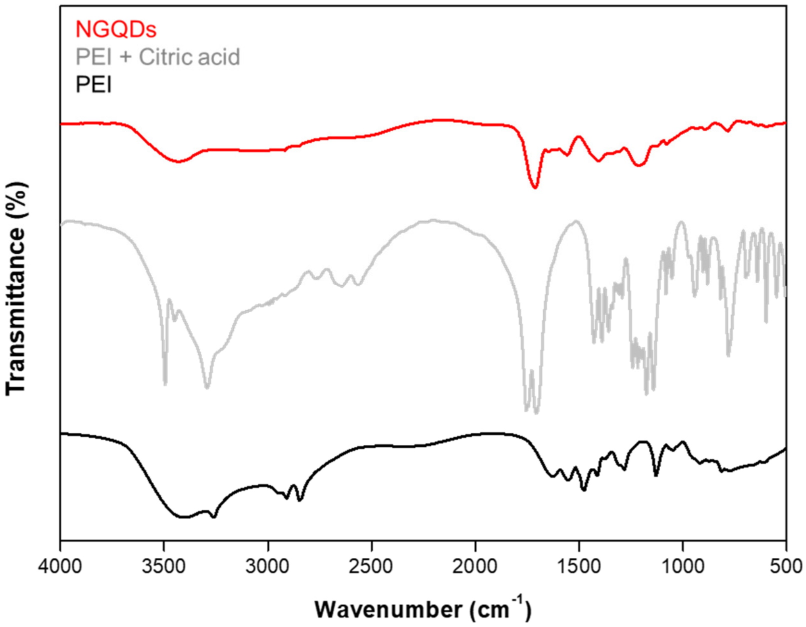

Figure S1. FT-IR spectra for NGQDs, PEI + citric acid, and PEI.

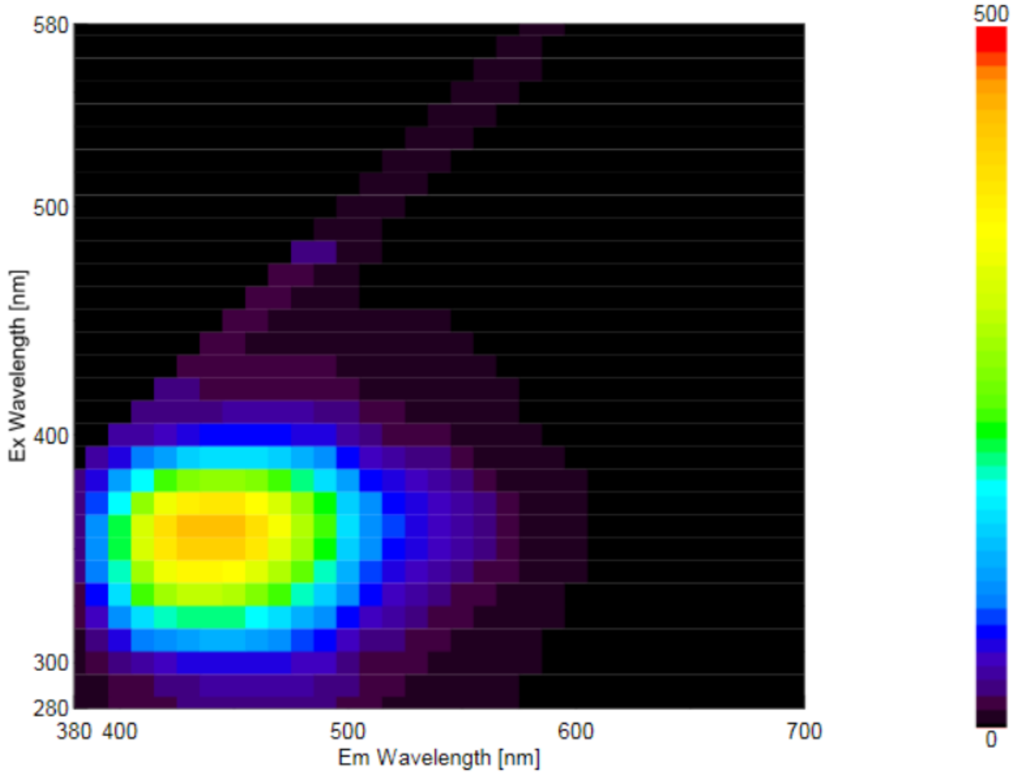

Figure S2. Emission spectra of NGQDs at excitation wavelength from 280 nm to 580 nm.

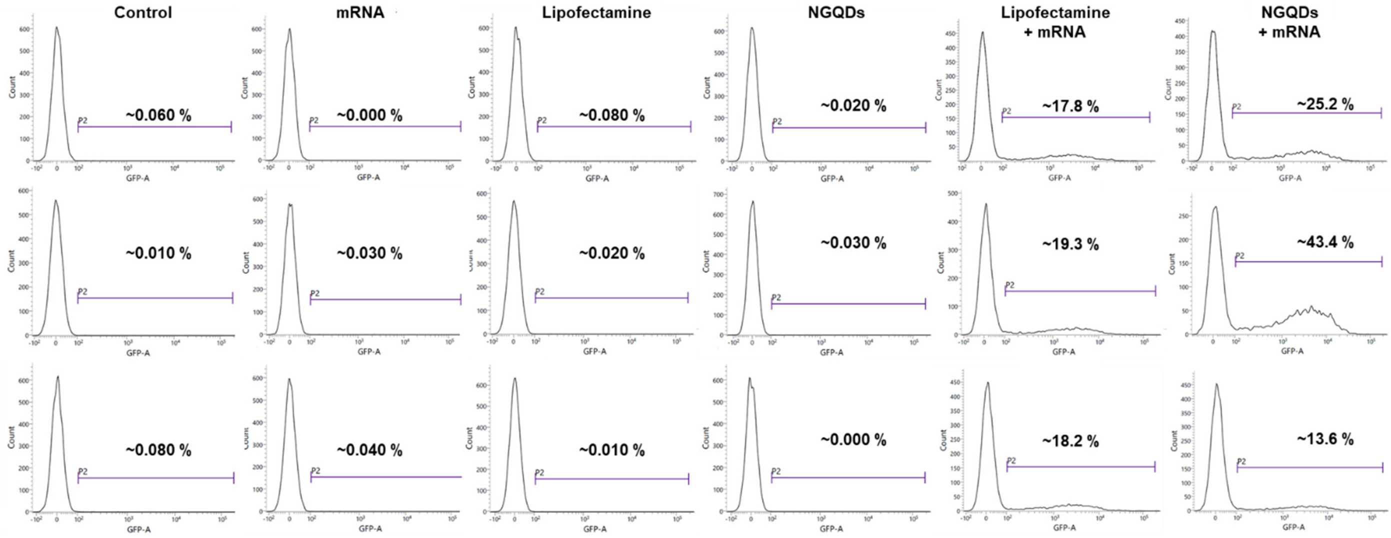

Figure S3. Flow cytometry analysis for mRNA transfection efficiency.

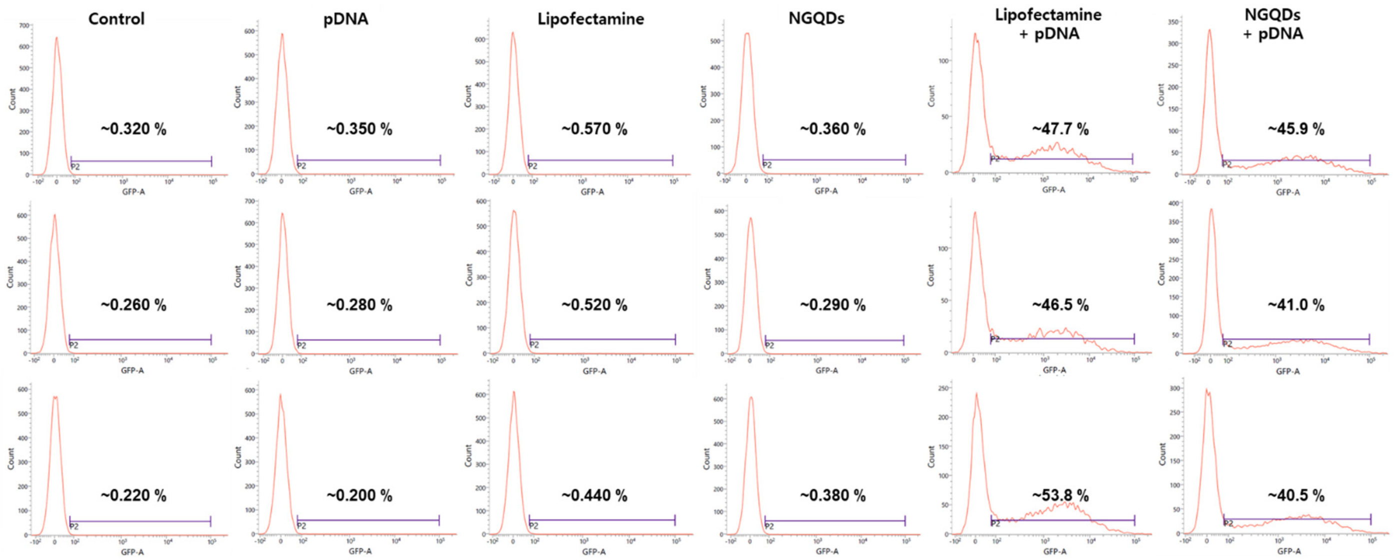

Figure S4. Flow cytometry analysis for pDNA transfection efficiency.
